# Designing a 2D van der Waals oxide with lone-pair electrons as chemical scissor

**DOI:** 10.1093/nsr/nwae370

**Published:** 2024-10-21

**Authors:** Zhipeng Du, Xu Chen, Wei Liu, Han Wang, Qianting Xu, Xiaoying Shang, Yipeng Song, Xueyuan Chen, Junhua Luo, Sangen Zhao

**Affiliations:** State Key Laboratory of Structural Chemistry, Fujian Institute of Research on the Structure of Matter, Chinese Academy of Sciences, Fuzhou 350002, China; Fujian College, University of Chinese Academy of Sciences, Fuzhou 350002, China; State Key Laboratory of Structural Chemistry, Fujian Institute of Research on the Structure of Matter, Chinese Academy of Sciences, Fuzhou 350002, China; Fujian College, University of Chinese Academy of Sciences, Fuzhou 350002, China; State Key Laboratory of Structural Chemistry, Fujian Institute of Research on the Structure of Matter, Chinese Academy of Sciences, Fuzhou 350002, China; State Key Laboratory of Structural Chemistry, Fujian Institute of Research on the Structure of Matter, Chinese Academy of Sciences, Fuzhou 350002, China; Fujian College, University of Chinese Academy of Sciences, Fuzhou 350002, China; State Key Laboratory of Structural Chemistry, Fujian Institute of Research on the Structure of Matter, Chinese Academy of Sciences, Fuzhou 350002, China; State Key Laboratory of Structural Chemistry, Fujian Institute of Research on the Structure of Matter, Chinese Academy of Sciences, Fuzhou 350002, China; Fujian College, University of Chinese Academy of Sciences, Fuzhou 350002, China; CAS Key Laboratory of Design and Assembly of Functional Nanostructures, and Fujian Key Laboratory of Nanomaterials, Fujian Institute of Research on the Structure of Matter, Chinese Academy of Sciences, Fuzhou 350002, China; State Key Laboratory of Structural Chemistry, Fujian Institute of Research on the Structure of Matter, Chinese Academy of Sciences, Fuzhou 350002, China; Fujian College, University of Chinese Academy of Sciences, Fuzhou 350002, China; State Key Laboratory of Structural Chemistry, Fujian Institute of Research on the Structure of Matter, Chinese Academy of Sciences, Fuzhou 350002, China; Fujian College, University of Chinese Academy of Sciences, Fuzhou 350002, China; CAS Key Laboratory of Design and Assembly of Functional Nanostructures, and Fujian Key Laboratory of Nanomaterials, Fujian Institute of Research on the Structure of Matter, Chinese Academy of Sciences, Fuzhou 350002, China; State Key Laboratory of Structural Chemistry, Fujian Institute of Research on the Structure of Matter, Chinese Academy of Sciences, Fuzhou 350002, China; Fujian College, University of Chinese Academy of Sciences, Fuzhou 350002, China; Fujian Science and Technology Innovation Laboratory for Optoelectronic Information of China, Fuzhou 350108, China; State Key Laboratory of Structural Chemistry, Fujian Institute of Research on the Structure of Matter, Chinese Academy of Sciences, Fuzhou 350002, China; Fujian College, University of Chinese Academy of Sciences, Fuzhou 350002, China; Fujian Science and Technology Innovation Laboratory for Optoelectronic Information of China, Fuzhou 350108, China; Quantum Science Center of Guangdong–Hong Kong–Macao Greater Bay Area (Guangdong), Shenzhen 518045, China

**Keywords:** 2D vdW materials, crystal structure, oxide, chemical scissor, second-harmonic generation

## Abstract

Two-dimensional (2D) van der Waals (vdW) materials are known for their intriguing physical properties, but their rational design and synthesis remain a great challenge for chemists. In this work, we successfully synthesized a new non-centrosymmetric oxide, i.e. InSbMoO_6_, with Sb^3+^ lone-pair electrons serving as chemical scissor to generate its 2D vdW crystal structure. Monolayer and few-layer InSbMoO_6_ flakes are readily obtained via facile mechanical exfoliation. They exhibit strong second-harmonic generation (SHG) response with an effective second-order nonlinear optical susceptibility $\chi _{{\mathrm{eff}}}^{{\mathrm{(2)}}}\ $of 32.4 pm·V^−1^. Meanwhile, the SHG response is in-plane anisotropic and directly proportional to the layer thickness, independent of layer parity. In addition, the InSbMoO_6_ flakes exhibit excellent thermal and atmospheric stability, along with pronounced anisotropy in Raman spectroscopy. This work implies that using lone-pair electrons as chemical scissor is an effective strategy for designing and synthesizing new 2D vdW materials for integrated photonic applications.

## Teaser

Lone-pair electron chemical scissors lead to a non-centrosymmetric 2D van der Waals oxide with strong in-plane anisotropy of nonlinear optical response.

## INTRODUCTION

Two-dimensional (2D) van der Waals (vdW) materials could provide an atomic-level flat interface so they facilitate integration without the constraints of lattice matching or processing [1,2]. They confine electrons within a plane and thus offer a low-dimensional platform for the exploration of on-chip optics and photonics across applications [3] including ultrathin quantum light sources [4], second harmonic generation (SHG) [5–9], polaritons [10], optoelectronics [11]. In contrast to isotropic 2D vdW materials [12], the utilization of in-plane anisotropic materials provides an additional degree of freedom for the tuning of electrical and optical properties, thereby expanding the range of possibilities for the design of innovative optical devices and the exploration of distinctive applications [13,14]. To date, observations have been made of several properties of 2D materials, including in-plane anisotropic thermal conductivity [15], polarization-resolved photoluminescence [16], anisotropic carrier mobility [17], polarization-resolved absorption [18], and polarization-resolved Raman [18]. Scientists have studied a series of anisotropic 2D materials, such as black phosphorus [19], transition metal dichalcogenides [20], *α*-MoO_3_ [21], MX_n_ (M = Ge, Sn; X = S, Se) [22–24], HfGe_0.92_Te [25], SiP_2_/MoS_2_ [26], SnP_2_Se_6_ [4], Penta-PdPSe [27], BN [28], NbOCl_2_ [4], and NbOI_2_ [6,29], which exhibit polarization dependence owing to their in-plane anisotropy. Nonetheless, these 2D materials either are unstable in air or show a layer-dependent SHG response [30,31]. Recently, we and co-authors found a series of tellurite molybdenum quaternary vdW oxides, including MgTeMoO_6_, ZnTeMoO_6_, MnTeMoO_6_, and CdTeMoO_6_ [32], as a highly promising 2D vdW family of materials for tunable low-loss anisotropic polariton. Analyzing these materials from a structural chemistry perspective, we find that the lone-pair electrons of Te^4+^ cations stop Te^4+^ from further coordination with neighboring oxygen atoms thereby forming 2D vdW structures [33]. These cations containing lone-pair electrons are called chemical scissors [34,35]. Therefore, we anticipate designing and synthesizing new 2D vdW materials by introducing cations with lone-pair electrons as chemical scissors.

In this work, we successfully designed and synthesized a new 2D vdW crystal, InSbMoO_6_ (ISM), using Sb^3+^ with 5s^2^ lone-pair electrons as a chemical scissor. We obtained monolayer ISM samples via mechanical exfoliation and conducted systematic investigations on their structure, stability, and SHG response.

## RESULTS AND DISCUSSION

**Figure 1.**
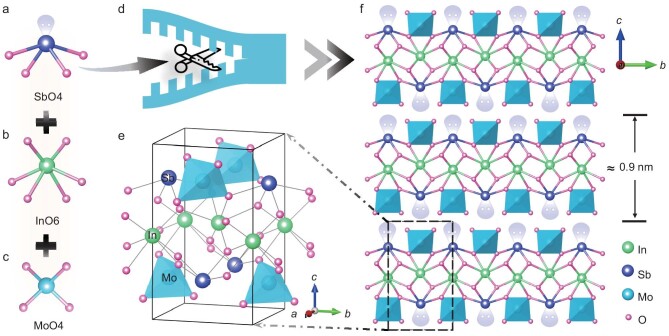
(a) SbO_4_ polyhedra with a stereochemically active lone pair, (b) InO_6_ octahedra, and (c) MoO_4_ polyhedra. (d) The lone-pair electrons in the SbO_4_ moiety serve as ‘chemical scissors’. (e) Schematic of the unit cell of ISM. (f) Top (out-of-plane) representations of the crystal structure of ISM. The blue polyhedra in the figure represent MoO_4_.

We synthesized polycrystalline ISM powders using a solid-state reaction. The raw materials used were In_2_O_3_, Sb_2_O_3_, and MoO_3_, mixed in a molar ratio of 1:1:2. Single crystals of ISM were grown using a spontaneous crystallization method inside sealed quartz tubes at 10 Pa and a temperature of 1050 K. Single-crystal X-ray diffraction (XRD) analysis determined the crystal structure of ISM, revealing its non-centrosymmetric space group *P*2_1_2_1_2. The cell parameters are *a* = 5.1634(3) Å, *b* = 5.3550(3) Å, *c* = 9.0067(6) Å, with *α* = *β* = *γ* = 90° and a volume of 249.04(3) Å^3^. Additional details are in Tables S1–S5 as well as the crystallographic information files (CIFs). The asymmetric unit of ISM consists of SbO_4_ (Fig. 1a), InO_6_ (Fig. 1b), and MoO_4_ (Fig. 1c). The lone-pair electrons in the SbO_4_ moiety serve as ‘chemical scissors’ because they prevent the Sb^3+^ cations from coordinating with neighboring oxygen atoms (Fig. 1d). As shown in Fig. 1e and 1f, SbO_4_ groups alternately occupy the up-side and down-side of two neighboring layers, making both layers merely packed via weak vdW interactions. The thickness of each layer is ∼0.9 nm (Fig. 1f). Each InO_6_ octahedron is corner-shared with four InO_6_ octahedra to form the 2D layered skeleton, and SbO_4_ polyhedra and MoO_4_ polyhedra are alternately linked to InO_6_ octahedra through corner-shared O atoms.

As shown in Fig. S1, the thermogravimetric (TG) and differential thermal analyses (DTA) reveal that ISM remains stable up to 1180 K, beyond which it decomposes (Fig. S2), as indicated by an endothermic peak. The powder XRD pattern closely matches the simulation based on the single-crystal structural data, confirming the phase purity of the ISM sample (Fig. S2). The UV-visible-near-infrared diffuse reflectance spectrum of ISM, measured between 300 nm and 800 nm (Fig. S3), shows the absorption edge at ∼427 nm in the UV spectral region. This absorption edge corresponds to an experimental bandgap of ∼2.9 eV. Fourier transform infrared spectroscopy (FTIR) of ISM, depicted in Fig. S4, reveals strong and sharp absorption peaks between 889 and 941 cm^−1^, attributed to Mo–O stretching vibrations. Additional peaks at 754 cm^−1^ and between 455 and 530 cm^−1^ are due to the stretching and wagging of In–O bonds, while signals between 628 and 666 cm^−1^ relate to the stretching and twisting modes of Sb–O bonds.

**Figure 2.**
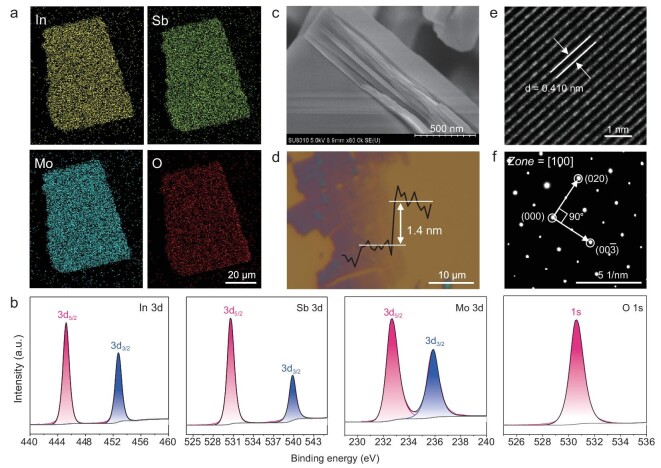
(a) Scanning electron microscope elemental mapping of a single crystal of ISM. The scale bar is set to 20 μm. (b) Fine XPS spectra of In 3d, Sb 3d, Mo 3d, and O 1s. (c) SEM image of the ISM. (d) AFM image of the monolayer ISM flake. (e) HAADF-STEM image and SAED pattern (f) of the ISM flake.

Scanning electron microscope elemental mapping shows an even distribution of In, Sb, Mo, and O elements across a single ISM crystal (Fig. 2a, Fig. S5). Additionally, X-ray photoelectron spectroscopy (XPS) was used to analyze the chemical composition of ISM. The survey scan confirmed the presence of In, Sb, Mo, and O elements (Fig. 2b). The fine XPS spectrum of In 3d, shown in Fig. 2b, displays two main peaks at ∼445.2 eV and 452.7 eV. These peaks are attributed to In–O bonds. The fine XPS spectrum of Sb 3d, with its deconvoluted peaks, is depicted in Fig. 2b. Peaks at 530.5 eV and 539.8 eV are attributed to Sb 3d. XPS analysis confirmed that Sb elements in the sample exist in the valence state of Sb^3+^, which typically possesses a lone pair of electrons. The peak at 530.6 eV in the O 1 s fine XPS spectrum is attributed to metal–O bonds. In the Mo 3d spectrum, peaks at 232.6 eV and 235.8 eV correspond to Mo 3d_5/2_ and Mo 3d_3/2_, respectively.

The layered structure of ISM crystals is clearly seen in Fig. 2c. Using the mechanical exfoliation method, regular monolayer flakes and large, rectangular, multi-layer ISM flakes with sharp edges (up to 25 μm in size, Fig. 2d, Fig. S6) are obtained. The specific atomic structure of the ISM flake, as verified by a high-angle annular dark-field scanning transmission electron microscope (HAADF-STEM), is shown in Fig. 2e and f. Fig. 2e presents a typical HAADF image of an ultrathin ISM flake, showing a crystal plane distance of 0.41 nm. Additionally, the STEM image and selected area electron diffraction (SAED) pattern (Fig. 2f) demonstrate that the ISM flake is a high-quality single crystal with a (100) top plane, belonging to the [100] crystal plane family. The sharp SAED spots indicate the exfoliated 2D ISM flake has high crystallinity.

**Figure 3.**
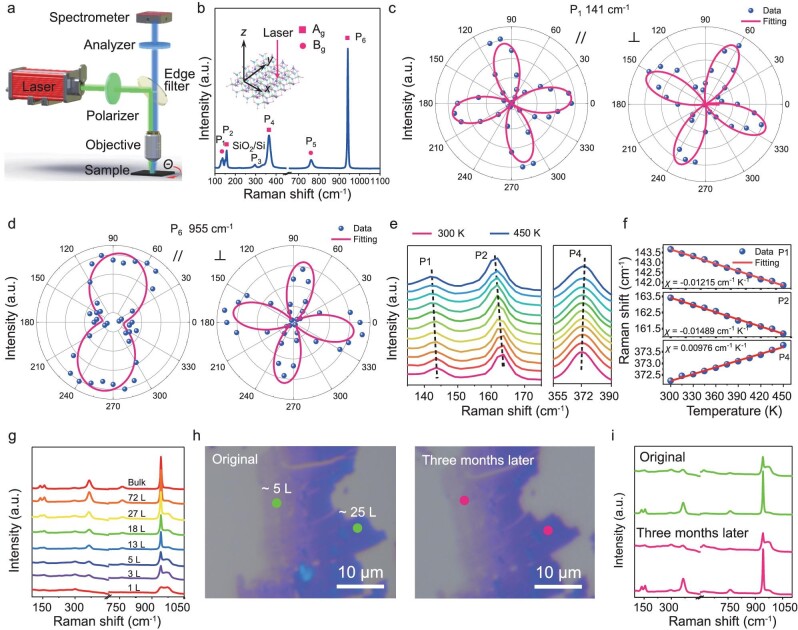
(a) Schematic diagrams of typical polarization configurations. (b) Raman spectra at the basal plane of ISM. The inset shows the schematic image of the basal plane for Raman measurement. Polar plots of the measured and fitted Raman peak intensities of the (c) P_1_ peak, and (d) P_6_ peak in parallel and perpendicular polarization configurations. (e) The detailed plots of offset in the P_1_, P_2_, and P_4_ peaks at different temperatures. Temperature-dependent Raman characterization. Raman spectra of 2D ISM flake measured at different temperatures ranging from 300 to 450 K. (f) Raman peak positions of P_1_, P_2_, and P_4_ as a function of temperature. (g) Raman spectra of 2D ISM flakes with different thicknesses. (h) Optical images and (i) Raman spectra of ISM flakes at different times.

The Raman spectrum provides comprehensive information about crystal structural orientation and phonon vibrations [36]. Angle-resolved polarized Raman (ARPR) spectroscopy was employed to determine Raman modes on the basis of crystal symmetry and selection rules, as well as to determine the crystallographic orientation of ISM flakes [29,30]. Due to its space group *P*2_1_2_1_2, the phonon vibrations of ISM are expected to be anisotropic. In this study, ARPR signals both parallel and perpendicular to the incident laser's polarization were collected from a rotating ISM flake (Fig. 3a). In Fig. 3b, P_1_ and P_6_ vibrations are mainly ascribed to Mo and O atoms, P_2_ is attributed to In and O atoms, P_4_ is the results of lattice vibrations, and P_5_ peak is mainly due to the vibrations of Mo, O, and Sb atoms, the Raman mode P_3_ peak is primarily attributed to the vibrational signature of SiO_2_/Si.

Fig. 3c, d and Fig. S7 illustrate that peaks P_1_ and P_5_ display a 4-lobed shape, while P_2_, P_4_, and P_6_ have a 2-lobed shape in parallel polarization configurations. Concurrently, all peaks manifest a 4-lobed shape in perpendicular polarization configurations. These findings clearly demonstrate that the polarized Raman intensities of ISM depend on its crystal structural orientation. Fig. 3c and d depict the polar plots of the P_x_ Raman positions in both parallel and perpendicular polarization configurations, where blue dots and red lines correspond to experimental values and fitting results from Raman tensor equations, respectively (Equations 4–7 in Supporting Information). The remaining three peaks are detailed in Fig. S7. The A_g_-like modes (P_2_, P_4_, and P_6_) exhibit 2-lobed and 4-lobed shapes in the parallel and perpendicular polarization configuration, respectively. The B_g_-like modes (P_1_ and P_5_) show a 4-lobed formation under the parallel and perpendicular polarization configuration. Significantly, A_g_-like modes exhibit maximum intensities along the axis in parallel configurations, aiding in determining the axial direction of 2D ISM flakes.

To examine the interlayer thermal expansion and atomic vibrations in the 2D ISM flake, we conducted temperature-dependent Raman characterization across a range of 300 to 450 K [29,37,38]. As depicted in Fig. S8a, Raman peak intensities rise as the temperature decreases, due to the enhanced anharmonic coupling of A_g_ and B_g_ modes at elevated temperatures. This coupling interaction conserves energy and reduces thermally vibrational excitation [29]. Fig. 3e and Fig. S8 illustrate the shift in Raman peaks across temperatures ranging from 300 to 450 K. The P_3_ peaks remain stable across temperatures, while P_1_ and P_2_ display a redshift as the temperature decreases, a consequence of anharmonic lattice vibrations at higher temperatures.

Fig. 3f further illustrates the temperature dependence of Raman peak positions. The temperature dependence of peak positions is described by the linear equation *ω*(*T*) = *ω*_0_ + *χ*(*T*), where *ω*_0_ is the Raman peak position at 0 K and *χ* represents the slope of the fitted line, corresponding to the first-order temperature coefficient of the Raman modes. The calculated *χ* values for P_1_, P_2_, and P_4_ are −0.01215, −0.01489, and 0.00978 cm^−1^·K^−1^, respectively. The temperature coefficient *χ* for ISM is larger than those observed in SnSe_2_ [39], RhI_3_ [40], and graphene [41]. The temperature coefficient of Raman modes is first-order linearly proportional to the interlayer forces of 2D vdW materials. Consequently, ISM exhibits stronger interlayer interactions than the above materials. This suggests that 2D ISM is more structurally stable under external forces, as compared to 2D materials with lower interlayer forces such as graphene. Fig. 3g shows that the Raman intensity of ISM flakes positively correlates with layer number, indicating strong interlayer interactions. As the number of layers decreases, ISM samples may exhibit special properties. The ISM flakes demonstrated excellent air stability at the 2D scale throughout the test period. After three months of exposure to air, no visible changes were observed in the ISM flakes (Fig. 3h). The thicknesses of the samples marked in the figure are 5 and 25 layers, respectively. The Raman spectra of the ISM flakes (Fig. 3i) remained consistent with their original condition. The significant thermal and air stability of ISM addresses the longstanding stability challenges faced by 2D materials like black phosphorus.

**Figure 4.**
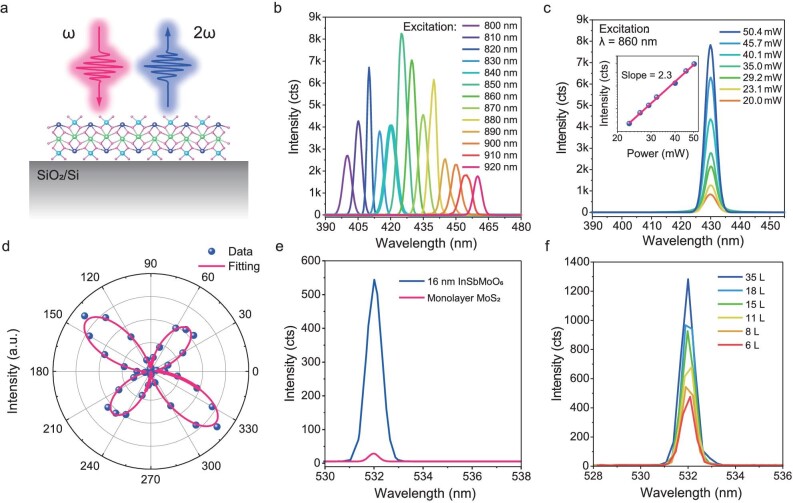
(a) Illustration of the measurement geometry. (b) SHG spectra of a 2D ISM flake at various excitation wavelengths ranging from 800 to 920 nm. (c) SHG spectra in the incident light of 860 nm with different power from 20.0 to 50.4 mW. Inset: pump-power-dependent SHG intensity. (d) Polarization-dependent SHG, indicating an anisotropic SHG response. (e) The SHG intensities of a 16 nm ISM flake and monolayer MoS_2_. (f) SHG spectra of layers with different thicknesses.

Nonlinear optical effects are crucial for studying non-centrosymmetric materials [42–45]. Given ISM's classification in the *P*2_1_2_1_2 space group with broken inversion symmetry, SHG was measured on a 2D ISM flake to explore associated nonlinear optical phenomena. The SHG response of the ISM was investigated in back-reflection experiments (Fig. 4a). Fig. 4b shows a strong SHG response from a 2D ISM flake across excitation wavelengths from 800 nm to 920 nm. Fig. 4b demonstrates a marked enhancement in SHG intensity as the wavelength shortens from ∼440 nm. Fig. 4c depicts how SHG intensities vary with power under an excitation wavelength of 860 nm. The generated laser wavelength is 430 nm, half of the value of the incident wavelength. Fig. S9 displays an optical image of the sample along with the SHG light spot. The inset in Fig. 4c shows SHG intensities in logarithmic coordinates, plotted against excitation power. The fitted plot's slope of 2.3, close to the theoretical value of 2, confirms that the signals primarily arise from SHG contributions. Fig. 4d shows that the SHG response is highly in-plane anisotropic, aligning well with crystal symmetry analysis [4]. In contrast to transition metal dichalcogenides, which show no polarization dependence in their SHG response [46,47], the total SHG intensity of ISM shows a strong azimuthal dependence on excitation, attributable to the in-plane anisotropy of crystals with low crystallographic symmetry. The pronounced orthorhombic SHG contrast of ISM facilitates easier identification of crystallographic orientations and applications in polarization-related second-order nonlinear phenomena, which surpass the capabilities of transition metal dichalcogenides [48–51].

To calculate the SHG conversion efficiency (*η*) of ISM flakes, monolayer MoS_2_ was used as a reference. Fig. 4e displays the SHG intensities for both monolayer MoS_2_ and 16 nm ISM flake. The refractive indices of ISM are 2.75 at 1064 nm and 2.91 at 532 nm, as shown in Fig. S10. Using the 16 nm ISM flake, the SHG intensity data suggests a second-order susceptibility ($\chi _{ISM}^{( 2 )}$) of ∼32.4 pm V^−1^ at 1064 nm, based on fundamental laser measurements [36]. The SHG efficiency of ISM, calculated as 21.3 times that of MoS_2_, results from comparing coefficients in the equation *η* = *P*_2ω_/*P*_ω_ = [8π^2^*d*^2^(*χ*^(2)^)^2^*P*_ω_]/[*n*_ω_^2^*n*2_ω_*λω*^2^*cε*_0_] [29,52]. Fig. 4f shows SHG signals collected from ISM flakes ranging from 6 to 35 layers under 1064 nm fundamental light. Contrary to the typical periodic decrease in SHG intensity with inversion symmetry, ISM shows an almost monotonically increasing trend, indicative of SHG signal enhancement irrespective of layer parity—an advantage of ISM in SHG application.

## CONCLUSIONS

In summary, by employing the strategy of “lone-pair electrons chemical scissors”, we discovered a stable 2D vdW oxide, InSbMoO_6_ (ISM). Using a mechanical stripping method, we have successfully obtained monolayer ISM samples. The ISM flakes demonstrate robust in-plane anisotropic Raman and SHG responses, with the SHG response being independent of layer parity. Additionally, they feature a significant effective second-order nonlinear susceptibility, $\chi _{{\mathrm{eff}}}^{{\mathrm{(2)}}}$ of ∼32.4 pm·V^−1^. These findings confirm that ISM is a promising air-stable, in-plane anisotropic nonlinear optical 2D material. Our findings provide an effective structural design strategy for developing 2D vdW crystals with potential integrated photonics applications.

## Supplementary Material

nwae370_Supplemental_Files
