# Supplementary material for: Designing a 2D van der Waals oxide with lone-pair electrons as chemical scissor
Source: Natl Sci Rev. 2024 Oct 21;12(1):nwae370. doi: 10.1093/nsr/nwae370 (PMC11702660; doi:10.1093/nsr/nwae370)
Supplement: nwae370_Supplemental_Files [file nwae370_supplemental_files.zip › 922 Supplementary_information.pdf]

Supplementary_information

Designing A 2D Van Der Waals Oxide with Lone Pair Electrons as Chemical Scissor

Zhipeng Du[1,4], Xu Chen[1,4], Wei Liu[1], Han Wang[1,4], Qianting Xu[1]*, Xiaoying Shang[1,2,4]*, Yipeng Song[1,4], Xueyuan Chen[1,2,4], Junhua Luo[1,4,5]*, Sangen Zhao[1,3,4,5]*

[1]State Key Laboratory of Structural Chemistry, Fujian Institute of Research on the Structure of Matter, Chinese Academy of Sciences, Fuzhou 350002, China

[2]CAS Key Laboratory of Design and Assembly of Functional Nanostructures, and Fujian Key Laboratory of Nanomaterials, Fujian Institute of Research on the Structure of Matter, Chinese Academy of Sciences, Fuzhou 350002, China

[3]Quantum Science Center of Guangdong–Hong Kong–Macao Greater Bay Area (Guangdong), Shenzhen 518045, China

[4]Fujian College, University of Chinese Academy of Sciences, Fuzhou 350002, China

[5]Fujian Science and Technology Innovation Laboratory for Optoelectronic Information of China, Fuzhou 350108, China

*Corresponding authors.

E-mails: zhaosangen@fjirsm.ac.cn; jhluo@fjirsm.ac.cn; shangxiaoying@fjirsm.ac.cn; QTXU19@163.com

Table of Contents

Experimental Procedures **1**
Synthesis of $InSbMoO_6$ (ISM) 1
Single-Crystal Structure Determination 1
Powder XRD Analysis 1
X-ray photoelectron spectroscopy (XPS) 2
Raman Analysis 2
Infrared Measurement 2
UV-vis-NIR Diffuse Reflectance Spectroscopy 3
Thermal Stability 3
SHG experiment 3
Results and Discussion **6**
Figure S1. TG-DTA curves of ISM. 6
Figure S2. Powder X-ray diffraction patterns of ISM. 6
Figure S3. The UV−vis−NIR diffuse reflectance spectrum of ISM. The inset indicates the experimental bandgap. 7
Figure S4. FTIR spectrum of ISM. 7
Figure S5. EDS Spectroscopy analysis for ISM. 8
Figure S6. The optical microscope images of the exfoliated ISM flakes. 8
Figure S7. Angle-resolved polarized Raman characterization. 9
Figure S8. Temperature-dependent Raman characterization. 10
Figure S9. Optical image of the sample and the SHG light (430 nm) spot. 11
Figure S10. Refractive index of ISM at the wavelengths from 100 to 1200 nm 11
Figure S11. The AFM scan profile of a original (left) and air-exposed five months later (right) flake. 12
Crystallographic data of ISM **13**
Table S1. Crystal Data and Structural Refinement Data for ISM. 13
Table S2. Fractional Atomic Coordinates ($\times 10^4$) and Equivalent Isotropic Displacement Parameters ($Å^2 \times 10^3$) for ISM. 14
Table S3. Anisotropic Displacement Parameters ($Å^2 \times 10^3$) for ISM. 14
Table S4. Bond Lengths for ISM. 14
Table S5. Bond Angles for ISM. 15
References **16**

Experimental Procedures

Synthesis of $InSbMoO_6$ (ISM)

All the reagents including In_2O_3 (Aladdin, 99.99%), Sb_2O_3 (Aladdin, 99.9%) and MoO_3 (Aladdin, 99.9%) are analytical grade from commercial sources without further purification.

In order to prevent the oxidation of Sb^{3+} under high temperature, single crystal of ISM was grown by the medium-temperature solid-state reaction method in closed system. A mixture of In_2O_3 (1 mmol) Sb_2O_3 (1 mmol) and MoO_3 (2 mmol) was ground thoroughly and loaded into separate graphite crucibles, and then put in a tidy quartz tube (Φ10 mm × 20 mm), which was flame-sealed under 10-20 Pa. The tube was heated to 550 °C in 6 h, and held at this temperature for 24 h, and then cooled to 350 °C with a rate of 1.0 °C/h, then cooled to room temperature. The millimeter-sized crystals were separated mechanically from the quartz glass tube.

Single-Crystal Structure Determination

Colorless block crystals of ISM were selected for the single-crystal structure determinations.[1] The diffraction data were collected at 212 K on a Rigaku XtaLAB Synergy-R diffractometer with graphite-monochromated Mo-Kα radiation (λ = 0.71073 Å). CrysAlisPro was employed to collect the intensity data, for cell refinement, and for crystal data reduction. Its structure was determined by a direct method on SHELXS and refined by SHELXL.[2] PLATON[3] verified that the determined structure was reasonable. Crystal data and structural refinement data are listed in Table S1 Crystallographic information, including atom isotropic or anisotropic displacement parameters and selected bond distances and angles, is summarized in Supporting Information Tables S2-S5.

Powder XRD Analysis

PXRD investigations on polycrystalline ISM were carried out at room temperature on a Rigaku MiniFlex 600 diffractometer equipped with Mo-Ka radiation. A scanning step width of 0.02° and scanning rate of 5° min^{-1} was applied to record the patterns in the 2θ range of 5-70°. The measured PXRD pattern matches well with the one calculated from single-crystal X-ray diffraction (XRD) analysis. The differences in

peak intensity for the same crystallographic index between the two patterns are believed to be caused by the preferential orientation of the powder samples.

X-ray photoelectron spectroscopy (XPS)

The X-ray photoelectron spectroscopy (XPS) was operated on the ESCALAB 250Xi XPS instrument by using Al Kα radiation as the source.

Raman Analysis

Raman spectroscopy between 100 and 1100 cm^{-1} range was collected on a LabRAM HR Evolution Raman microscope (HORIBA Scientific) with a solid-state laser corresponding to the green light (λ = 532 nm).

The intensity of observed Raman vibration modes can be expressed as[4]:

$$I \propto |e_i R e_s|^2 \quad (1)$$

where e_i and e_s are the unit polarization vectors of incident and scattered lasers respectively, and R is the Raman tensor. The unit polarization vector $e_i = (cos\theta, sin\theta, 0)$, where θ is the angle between incident light polarization and a axis direction of the ISM. Similarly, under the parallel configuration, e_s can be written as $e_s = (cos\theta, sin\theta, 0)$, while in the perpendicular configuration, it can be written as $e_s = (-sin\theta, cos\theta, 0)$. Since ISM crystallizes in $P2_12_12$ space group, the Raman tensor can be written as

$$R(A_g) = \begin{bmatrix} a & 0 & 0 \\ 0 & b & 0 \\ 0 & 0 & c \end{bmatrix} \quad (2)$$

$$R(B_g) = \begin{bmatrix} 0 & d & 0 \\ d & 0 & 0 \\ 0 & 0 & 0 \end{bmatrix} \quad (3)$$

Hence, the anisotropy of Raman scattering intensities in the parallel and perpendicular polarization configurations can be expressed by the following equations[5]:

$$I(A, \parallel) \propto cos^2\theta + sin^2\theta \quad (4)$$

$$I(B, \parallel) \propto sin^2 2\theta \quad (5)$$

$$I(A, \perp) \propto sin^2 2\theta \quad (6)$$

$$I(B, \perp) \propto cos^2 2\theta \quad (7)$$

Infrared Measurement

The Fourier transform infrared spectroscopy (FT-IR) spectrum of ISM was recorded on a Bruker VERTEX 70 infrared spectrometer at room temperature in the wavenumber from 400 to 4000 cm^{-1}.

UV-vis-NIR Diffuse Reflectance Spectroscopy

The UV-vis-NIR diffuse reflection data were recorded at room temperature using a powdered $BaSO_4$ sample as a standard (100% reflectance) on a PerkinElmer Lamda-950 UV/vis/NIR spectrophotometer. The scanning wavelength range is from 300 nm to 800 nm. Absorption (*K/S*) data were calculated from the following Kubelka-Munk function[6]:

$$F(R) = \frac{(1-R)^2}{2R} = \frac{K}{S} \qquad (8)$$

where *R* is the reflectance, *K* is the absorption, and *S* is the scattering. In the (*K/S*) versus *F(R)* plot, extrapolating the linear portion of the rising curve to zero gives rise to the onset of absorption. The experimental band gap is obtained.

Thermal Stability

The thermal stability of ISM was simultaneously investigated using a NETZSCH STA 449F3 thermal analyzer (the DTA was calibrated with Al_2O_3). About 15 mg of ISM was placed in an Al_2O_3 crucible, and heated at a rate of 10 K min^{-1} from room temperature to 1440 K under flowing nitrogen gas. Thermogravimetric and differential thermal analyses curves reveal that ISM keeps stable below 1180 K, and then ISM will decompose, as indicated by the corresponding endothermic peak.

SHG experiment

The SHG experiments were conducted on a home-built optical system operated in reflection geometry. A tunable femtosecond laser (pulse width: 70 fs; repetition frequency: 80 MHz; 710-920 nm, Mai Tai XF-1, Spectra-Physics) was used as the excitation source, which passes through a polarizing beamsplitter, an 800 nm half-wave plate, a dichroic mirror and an objective (50×, NA 0.8, Nikon) before interaction with samples. The same objective was used to collect the SHG signal. The

spectrum was measured using a spectrometer (Princeton Instruments, SP2500) cooled by liquid nitrogen. All of the experiments were carried out at room temperature.

Calculation of Second-order nonlinear susceptibility

$$P_{2\omega} = \frac{8\pi^2 d^2}{\varepsilon_0 c \lambda^2 A} \cdot \frac{\left[\chi^{(2)}\right]^2}{n_\omega^2 n_{2\omega}} \cdot P_\omega^2 \quad (9)$$

Where P_ω and $P_{2\omega}$ are the power of excitation laser and SHG signal, respectively. $\chi^{(2)}$ is the second-order susceptibility. Efficiency of signal collection and detection, we can estimate the optical second-order susceptibility of ISM in contrast with monolayer ${\chi_{MoS2}}^{(2)}$ which had been measured several times[7-11]. ε_0 is the vacuum dielectric constant. A is the illuminance area, c is the speed of light in vacuum. and are the refractive index of MoS_2 at frequency 2ω of excitation laser and at frequency ω of SHG field, respectively. And d is the thickness of the flakes. We cannot directly measure the power of the SHG signal and this formula also can applies to MoS_2, under the same test conditions, with a reasonable consideration of the efficiency of signal collection and detection, we can estimate the optical second-order susceptibility of ISM in contrast with monolayer which had been measured several times.

$$\frac{\chi_{ISM}^{(2)}}{\chi_{MoS_2}^{(2)}} = \left(\frac{P_{2\omega-ISM}}{P_{2\omega-MoS_2}}\right)^{\frac{1}{2}} \cdot \frac{d_{MoS_2}}{d_{ISM}} \cdot \left(\frac{n_{2\omega-ISM} n_{\omega-ISM}^2}{n_{2\omega-MoS_2} n_{\omega-MoS_2}^2}\right)^{\frac{1}{2}} \quad (10)$$

Under the same test conditions, like the same excitation wavelength, power, test environment, we can equivalent to equation (4) by equation (3). The SHG signal power is proportional to the intensity, we can came to equation (4) and get the ratio relationship between $\chi_{ISM}^{(2)}$ and $\chi_{MoS_2}^{(2)}$. The second harmonic generation intensity from CVD monolayer MoS_2 and 16 nm ISM flake under the same test conditions. When the excitation laser wavelength is 1064 nm, $n_{2\omega-ISM}$ =2.91 and $n_{\omega-ISM}$ =2.75, $n_{2\omega-MoS_2}$=3.8 and $n_{\omega-MoS_2}$=4.4, respectively. We can estimate the optical second-order susceptibility of ISM is $0.08\chi_{MoS_2}^{(2)}$. Liu et al. have estimated the monolayer the optical second-order susceptibility of $\chi_{MoS_2}^{(2)}$ is 405 pm/V[11], This approach leads to $\chi_{ISM}^{(2)}$=32.4 pm/V for the 16 nm ISM flake.

SHG conversion efficiency is another important parameter to measure its nonlinear performance and can be expressed as

$$\eta = \frac{P_{2\omega}}{P_\omega} = \frac{8\pi^2 d^2}{\varepsilon_0 c \lambda^2 A} \cdot \frac{\left[\chi^{(2)}\right]^2}{n_\omega^2 n_{2\omega}} \cdot P_\omega \quad (11)$$

Similarly, we can estimate the SHG conversion efficiency of ISM in contrast with monolayer $\chi^{(2)}_{MoS_2}$, η_{ISM}can be expressed as

$$\eta_{ISM} = \frac{d_{ISM}^2(\chi_{ISM}^{(2)})^2 n_{2\omega-MoS_2} n_{\omega-MoS_2}^2}{d_{MoS_2}^2(\chi_{MoS_2}^{(2)})^2 n_{2\omega-ISM} n_{\omega-ISM}^2} \cdot \eta_{MoS_2} \quad (12)$$

This approach leads to η_{ISM}=21.3η_{MoS_2}=2.13%[11]. The value of $\chi^{(2)}$ and η rely on accurate knowledge of many experimental parameters, such as frequency and duration of the excitation pulse, the shape and size of the focused fundamental spot at the sample, and the relation between the measured spectral counts and the actual SH power. Hence, the values should be viewed as an order of magnitude estimate for reference.

Results and Discussion

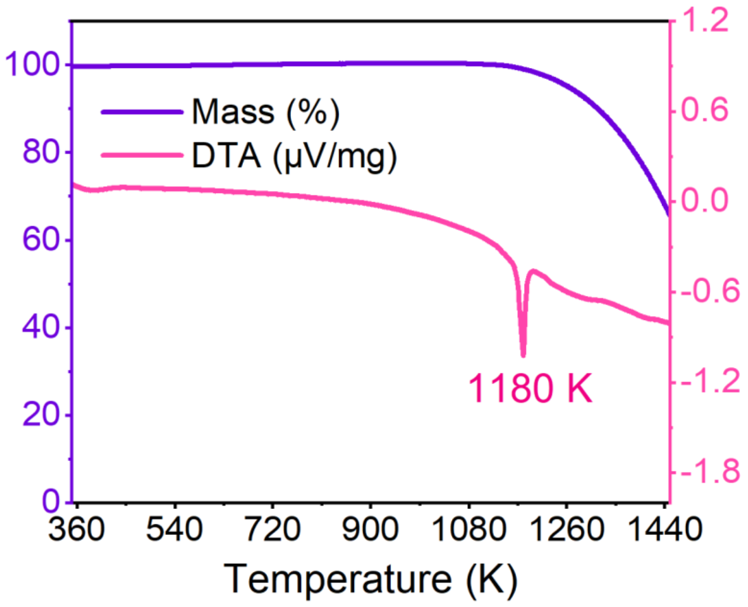

Figure S1. TG-DTA curves of ISM.

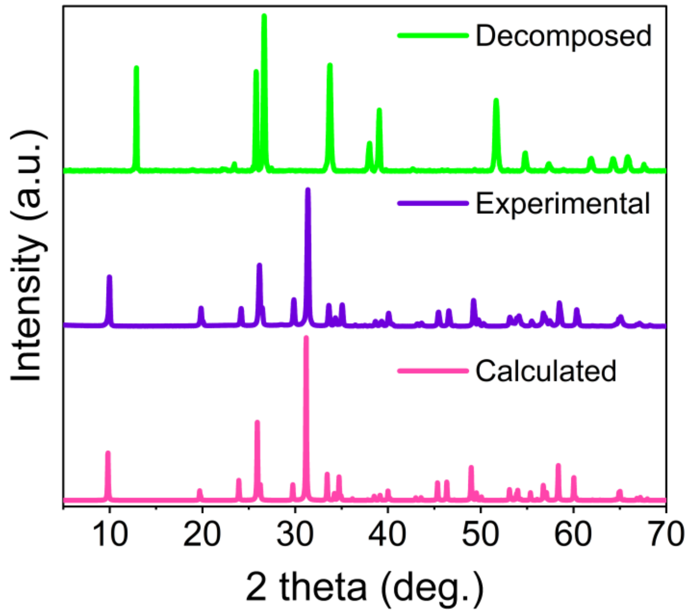

Figure S2. Powder X-ray diffraction patterns of ISM.

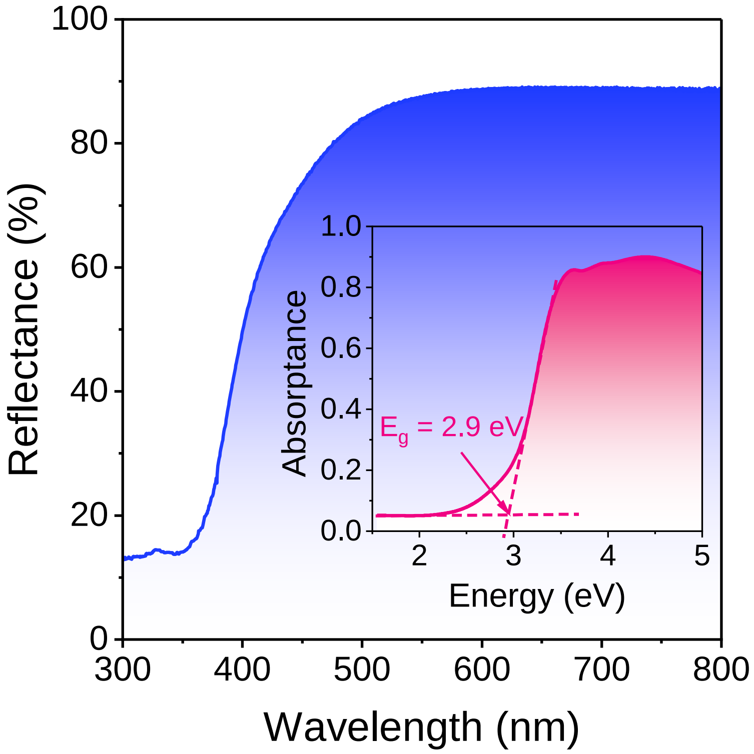

Figure S3. The UV−vis−NIR diffuse reflectance spectrum of ISM. The inset indicates the experimental bandgap.

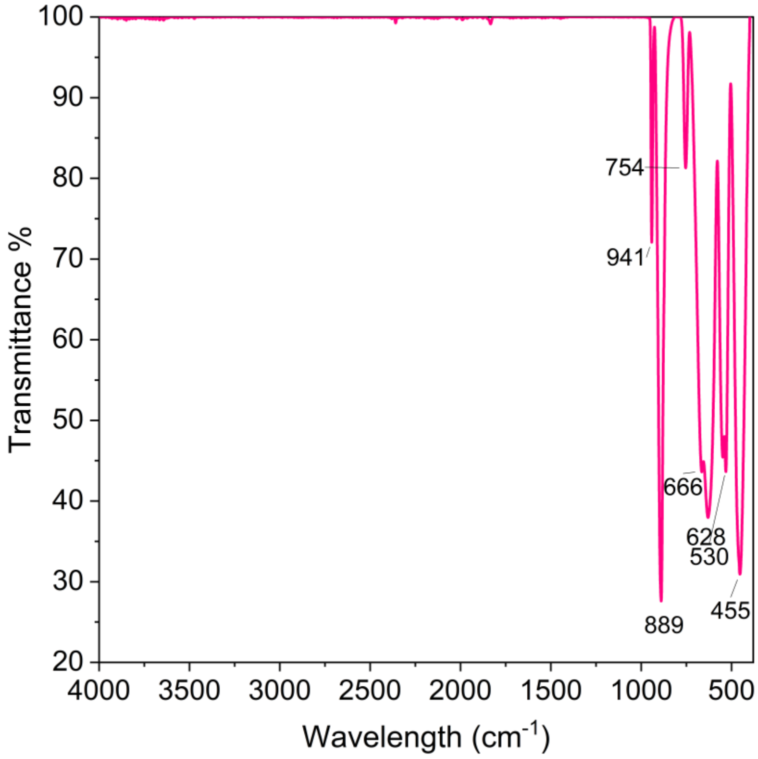

Figure S4. FTIR spectrum of ISM.

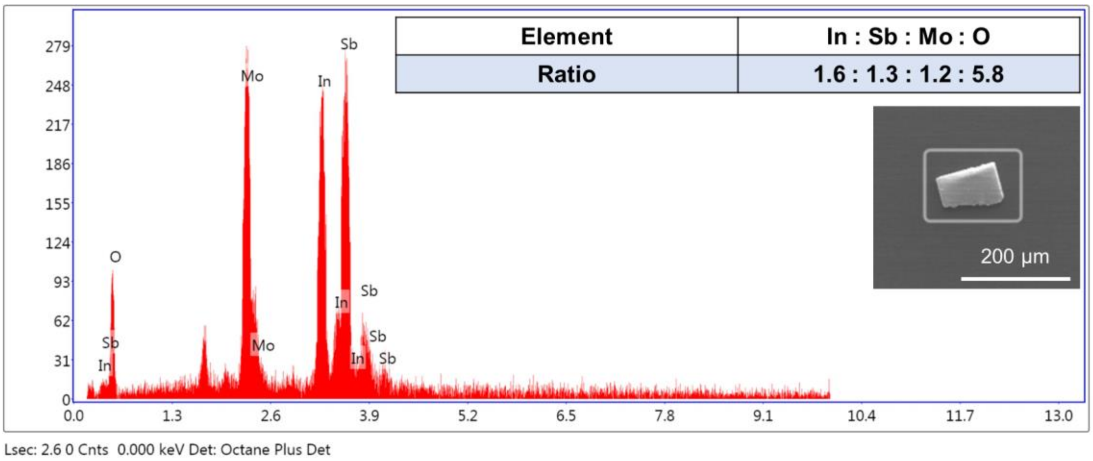

Figure S5. EDS Spectroscopy analysis for ISM.

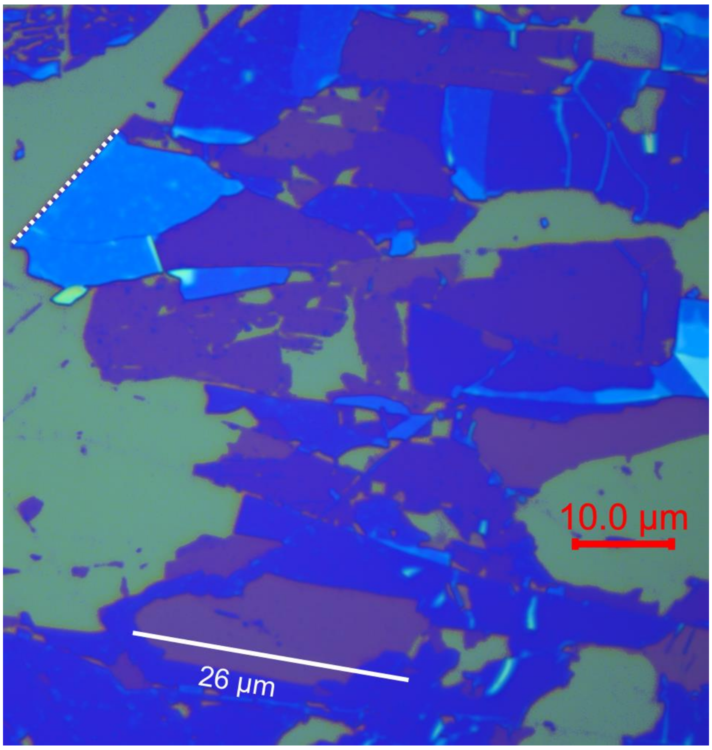

Figure S6. The optical microscope images of the exfoliated ISM flakes.

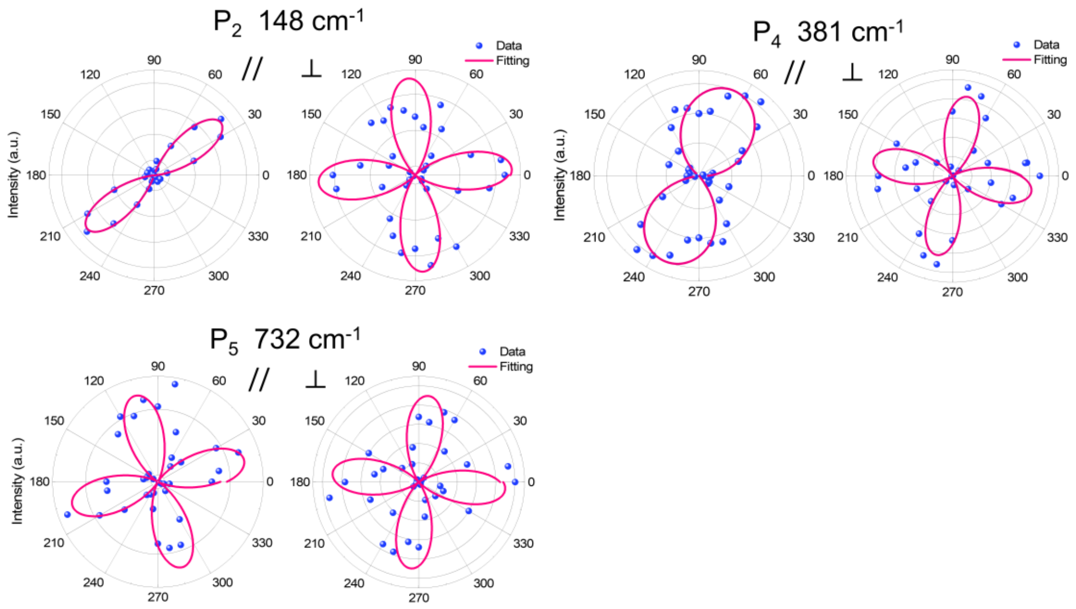

Figure S7. Angle-resolved polarized Raman characterization. Polar plots of the measured and fitted Raman peak intensities of the P_2 peak, P_4 peak, and P_5 peak in parallel and perpendicular polarization configurations.

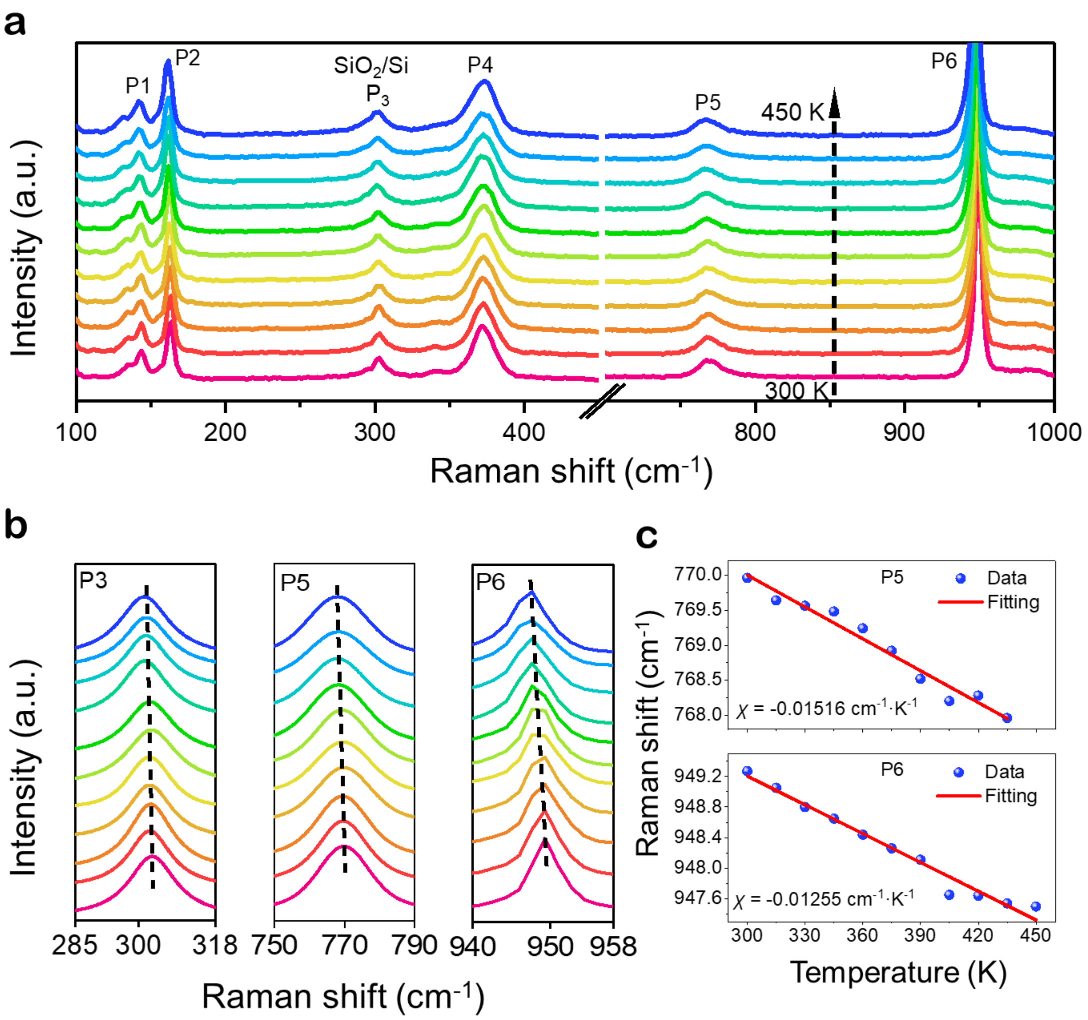

Figure S8. Temperature-dependent Raman characterization. **a**. Raman spectra of 2D $InSbMoO_6$ flake measured at different temperatures ranging from 300 to 450 K. **b**. The detailed plots of offset in the P3, P5, and P6 peaks at different temperature. **c**. Raman peak positions of P5, and P6 as a function of temperature.

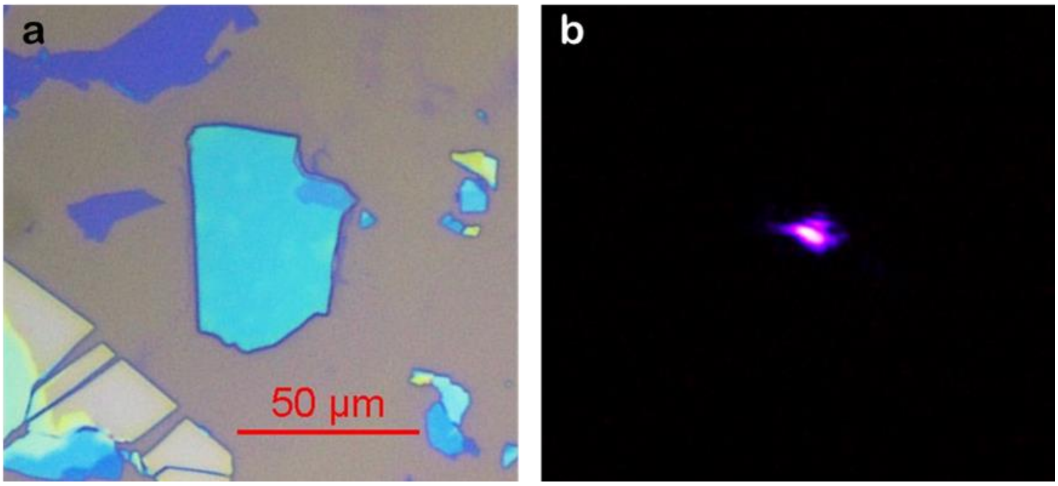

Figure S9. Optical image of the sample (a) and the SHG light (430 nm) spot (b).

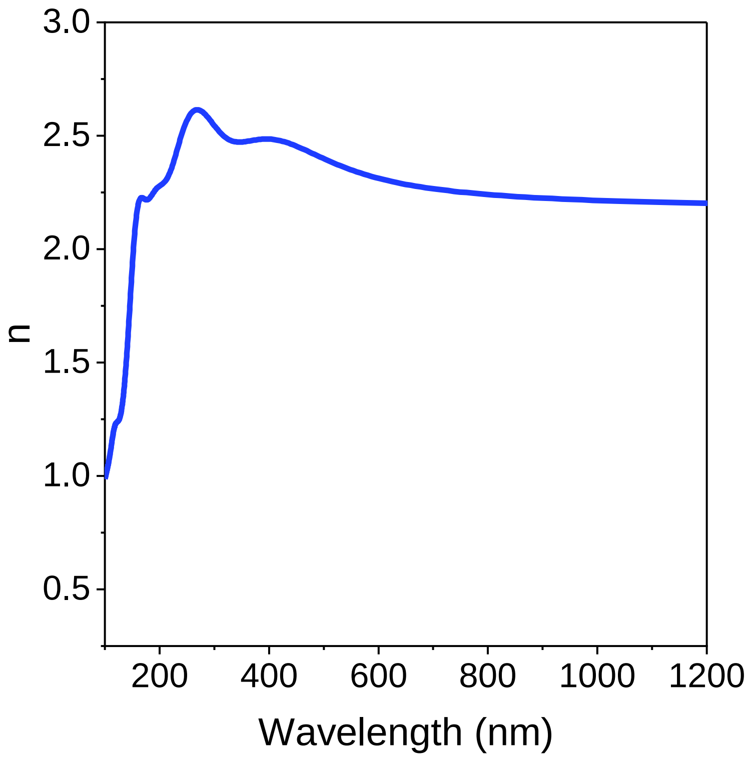

Figure S10. Refractive index of ISM at the wavelengths from 100 to 1200 nm

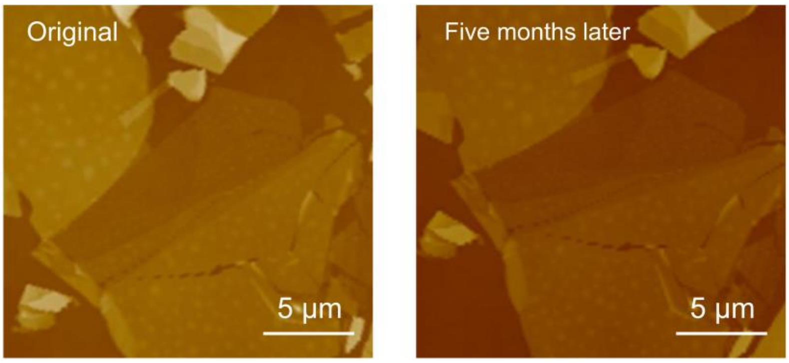

Figure S11. The AFM scan profile of an original (left) and air-exposed five months later (right) flake.

Crystallographic data of ISM

Table S1. Crystal Data and Structural Refinement Data for ISM.

Empirical formula	$InSbMoO_6$
Formula weight/g/mol	428.51
Temperature/K	212
Crystal system	Orthorhombic
Space group	$P2_12_12$
a/Å	5.1634(3)
b/Å	5.3550(3)
c/Å	9.0067(6)
$\alpha=\beta=\gamma$/ °	90
Volume/Å^3	249.04(3)
Z	2
ρ_{calc}/g/cm^3	5.715
μ/mm^{-1}	12.404
F(000)	380.0
Radiation	Mo Kα (λ = 0.71073)
h, k, l max	5, 6, 10
2Θ range for data collection/ °	8.856 – 50.038
Independent reflections	431 [R_{int} = 0.046, R_{sigma} = 0.0302]
Goodness-of-fit on F^2	1.000
Final R indices [I>2σ(I)][a]	R_1 = 0.0234, wR_2 = 0.0588
Final R indexes [all data]	R_1 = 0.0259, wR_2 = 0.0601
Largest diff. peak/hole / e Å^{-3}	1.85/-0.70
Flack parameter	0.20(9)

[a] $R_1 = \sum||F_O|-|F_C||/\sum|F_O|$ and $wR_2 = [\sum[w(F_O^2-F_C^2)^2]/\sum[w(F_O^2)^2]]^{1/2}$ for $F_O^2 > 2\sigma(F_C^2)$

Table S2. Fractional Atomic Coordinates (×10^4) and Equivalent Isotropic Displacement Parameters ($Å^2$ ×10^3) for ISM.

Atom	*x*	*y*	*z*	*U(eq)**
Sb01	0	5000	2364.9(8)	11.9(3)
In02	5000	5000	5219.5(9)	12.2(3)
Mo03	5000	10000	1944.4(11)	13.1(3)
O004	2529(12)	8220(13)	2967(8)	15.4(14)
O005	2148(13)	3217(13)	3894(7)	13.7(14)
O006	6523(12)	7963(13)	789(9)	20.6(16)

*U_{eq} is defined as 1/3 of the trace of the orthogonalised U_{ij} tensor.

Table S3. Anisotropic Displacement Parameters ($Å^2$ ×10^3) for ISM.

Atom	U_{11}	U_{22}	U_{33}	U_{23}	U_{13}	U_{12}
Sb01	8.6(5)	9.9(5)	17.3(5)	0	0	1.7(7)
In02	7.6(5)	9.1(5)	19.9(5)	0	0	-0.4(6)
Mo03	8.2(6)	13.0(6)	18.0(6)	0	0	-4.3(9)
O004	12(3)	6(3)	27(4)	-5(3)	6(3)	2(3)
O005	12(3)	6(3)	23(4)	2(3)	-4(3)	-2(3)
O006	11(3)	20(4)	30(4)	-5(3)	-2(3)	6(3)

The Anisotropic displacement factor exponent takes the form: $-2\pi^2[h^2a^{*2}U_{11}+2hka^*b^*U_{12}+...]$.

Table S4. Bond Lengths for ISM.

Atom	Atom	Length/Å	Atom	Atom	Length/Å
Sb01	O004	2.230(7)	In02	O005[5]	2.122(6)
Sb01	O004[1]	2.230(7)	In02	O005[6]	2.199(6)
Sb01	O005[1]	2.010(6)	In02	O005	2.122(6)
Sb01	O005	2.010(6)	Mo03	O004[7]	1.840(7)
In02	O004[2]	2.298(6)	Mo03	O004	1.840(7)
In02	O004[3]	2.298(6)	Mo03	O006	1.700(7)
In02	O005[4]	2.199(6)	Mo03	O006[7]	1.700(7)

[1]-X,1-Y,+Z; [2]1/2-X,-1/2+Y,1-Z; [3]1/2+X,3/2-Y,1-Z; [4]1/2+X,1/2-Y,1-Z; [5]1-X,1-Y,+Z; [6]1/2-X,1/2+Y,1-Z; [7]1-X,2-Y,+Z

Table S5. Bond Angles for ISM.

Atom1	Atom2	Atom3	Angle/°	Atom1	Atom2	Atom3	Angle/°
O004	Sb01	O004^{1}	151.9(4)	O005^{6}	In02	O005^{5}	101.92(16)
O005	Sb01	O004^{1}	77.8(2)	O005^{6}	In02	O005	111.6(4)
O005^{1}	Sb01	O004	77.8(2)	O005^{4}	In02	O005^{5}	137.4(3)
O005^{1}	Sb01	O004^{1}	83.0(3)	O005	In02	O005^{5}	101.63(14)
O005	Sb01	O004	83.0(3)	O004^{7}	Mo03	O004	119.9(4)
O005^{1}	Sb01	O005	93.5(4)	O006	Mo03	O004^{7}	108.6(3)
O004^{2}	In02	O004^{3}	89.4(3)	O006^{7}	Mo03	O004^{7}	107.1(3)
O005^{4}	In02	O004^{2}	72.8(2)	O006	Mo03	O004	107.1(3)
O005^{5}	In02	O004^{2}	77.3(3)	O006^{7}	Mo03	O004	108.6(3)
O005	In02	O004^{3}	79.6(2)	O006	Mo03	O006^{7}	104.5(5)
O005^{6}	In02	O004^{3}	168.7(3)	Sb01	O004	In02^{4}	99.2(3)
O005^{5}	In02	O004^{3}	72.8(2)	Mo03	O004	Sb01	133.2(4)
O005^{4}	In02	O004^{3}	77.3(3)	Mo03	O004	In02^{4}	122.3(3)
O005	In02	O004^{2}	168.7(3)	Sb01	O005	In02^{3}	110.0(3)
O005^{6}	In02	O004^{2}	79.6(2)	Sb01	O005	In02	123.7(3)
O005	In02	O005^{4}	101.92(16)	In02	O005	In02^{3}	119.9(3)
O005^{6}	In02	O005^{4}	101.63(14)				

^{1}-X,1-Y,+Z; ^{2}1/2+X,3/2-Y,1-Z; ^{3}1/2-X,-1/2+Y,1-Z; ^{4}1/2-X,1/2+Y,1-Z; ^{5}1/2+X,1/2-Y,1-Z; ^{6}1-X,1-Y,+Z; ^{7}1-X,2-Y,+Z

References

[1] Zhou, Y.; Li, Y.; Ding, Q.; Liu, Y.; Chen, Y.; Liu, X.; Huang, X.; Li, L.; Zhao, S.; Luo, J. Noncentrosymmetric $K_2Mn_3(SO_4)_3F_2 \cdot 4H_2O$ and $Rb_2Mn_3(SO_4)_3F_2 \cdot 2H_2O$ with pseudo-KTP structures. *Chinese Chemical Letters* **2021**, *32* (1), 263-265.

[2] Berman, H. M. The protein data bank: a historical perspective. *Acta Crystallographica Section A* **2008**, *64* (1), 88-95.

[3] Van der Sluis, P.; Spek, A. BYPASS: an effective method for the refinement of crystal structures containing disordered solvent regions. *Acta Crystallographica Section A: Foundations of Crystallography* **1990**, *46* (3), 194-201.

[4] Loudon, R. The Raman Effect in Crystals. Adv. Phys. 1964, 13, 423–482.

[5] H. B. Ribeiro, M. A. Pimenta, C. J. S. de Matos, R. L. Moreira, A. S. Rodin, J. D. Zapata, E. A. T. de Souza, A. H. Castro Neto, ACS Nano 2015, 9, 4270.

[6] Tauc, J. Absorption edge and internal electric fields in amorphous semiconductors. Mater. Res. Bull. 1970, 5 (8), 721-729.

[7] D. J. Clark, V. Senthilkumar, C. T. Le, D. L. Weerawarne, B. Shim, J. I. Jang, J. H. Shim, J. Cho, Y. Sim, M. J. Seong, S. H. Rhim, A. J. Freeman, K. H. Chung, Y. S. Kim, Phys. Rev. B 2014, 90, 121409.

[8] N. Kumar, S. Najmaei, Q. Cui, F. Ceballos, P. M. Ajayan, J. Lou, H. Zhao, Phys. Rev. B 2013, 87, 161403.

[9] Y. Li, Y. Rao, K. F. Mak, Y. You, S. Wang, C. R. Dean, T. F. Heinz, Nano Lett. 2013, 13, 3329.

[10] L. M. Malard, T. V. Alencar, A. P. M. Barboza, K. F. Mak, A. M. Paula, Phys. Rev. B 2013, 87, 201401.

[11] J. Shi, P. Yu, F. Liu, P. He, R. Wang, L. Qin, J. Zhou, X. Li, J. Zhou, X. Sui, S. Zhang, Y. Zhang, Q. Zhang, T. C. Sum, X. Qiu, Z. Liu, X. Liu, Adv. Mater. 2017, 29, 1701486.
